# Use of ultrasonography in confirmation of endotracheal tube position

**DOI:** 10.1186/2197-425X-3-S1-A937

**Published:** 2015-10-01

**Authors:** A Abdalla, HF Rida, EM Khalil

**Affiliations:** Faculty of Medicine, Critical Care, Alexandria University, Alexandria, Egypt; Faculty of Medicine, Anaesthesia and Surgical Intensive Care, Alexandria University, Alexandria, Egypt

## Introduction

Ultrasound (US) imaging technique has recently emerged as a novel, simple, portable and non invasive tool helpful for airway assessment and management. It helps in rapid assessment of airway anatomy, not only in operation theatre but also in the intensive care unit and emergency department.

## Objectives

The aim of this study was to evaluate the ability of ultrasonography to confirm the correct position of endotracheal tube (main stem of the trachea).

## Methods

This study was conducted on 100 patients who were admitted to Critical Care Medicine Department in Alexandria Main University Hospital with an indication of endotracheal tube placement. All patients were examined by ultrasonography "linear probe" after endotracheal tube insertion. Results were compared with capnometric readings (end tidal CO_2_). After confirmation of endotracheal tube position (not esophageal), all ETTs were re-examined by ultrasonography after inflation of cuffs of ETT by 10 ml saline. X-ray identification of the position of the distal tips of ETTs confirmed whether the ETT was above the suprasternal notch (above the carina) or below.

## Results

Use of ultrasonography to detect the position of the endotracheal tube (tracheal or esophageal) in both the horizontal and vertical views at the anatomical tracheal site in comparison to capnometry revealed: - sensitivity, specificity, positive predictive value, negative predictive value and accuracy were 98.96%, 100%, 100%, 80% and 99.0% respectively, The ROC curve showed AUC (area under the curve) was 0.995. Results of ultrasonographic image of the cuff of ETTs in comparison to X-ray revealed: - sensitivity, specificity, positive predictive value, negative predictive value and accuracy were 82.76%, 100%, 100%, 46.43% and 85% respectively, The ROC curve showed AUC (area under the curve) was 0.914. The ultrasonographic duration to detect the endotracheal tube whether tracheal or esophageal ranged from 4 to 16 seconds with a mean (± SD) of 8.61 ± 2.66 and a median of 8.50 seconds

## Conclusions

Ultrasonography can be used not only to detect endotracheal tube position in the trachea not in the oesophagus but also to detect the position of ETT inside the trachea" above suprasternal notch or below (above carina not in the right main bronchus)
